# Supplementary material for: The Membrane Proteome of Spores and Vegetative Cells of the Food-Borne Pathogen Bacillus cereus
Source: Int J Mol Sci. 2021 Nov 19;22(22):12475. doi: 10.3390/ijms222212475 (PMC8624511; doi:10.3390/ijms222212475)
Supplement: Supplementary file 1 [file ijms-22-12475-s001.zip › ijms-1446819-supplementary/Supplemental figures.pdf]

The membrane proteome of spores and vegetative cells of the food-borne pathogen Bacillus cereus.

Xiaowei Gao[1,2], Bhagyashree N. Swarge[1,2], Henk L. Dekker[2], Winfried Roseboom[2], Stanley Brul[1,*] and Gertjan Kramer[2,*]

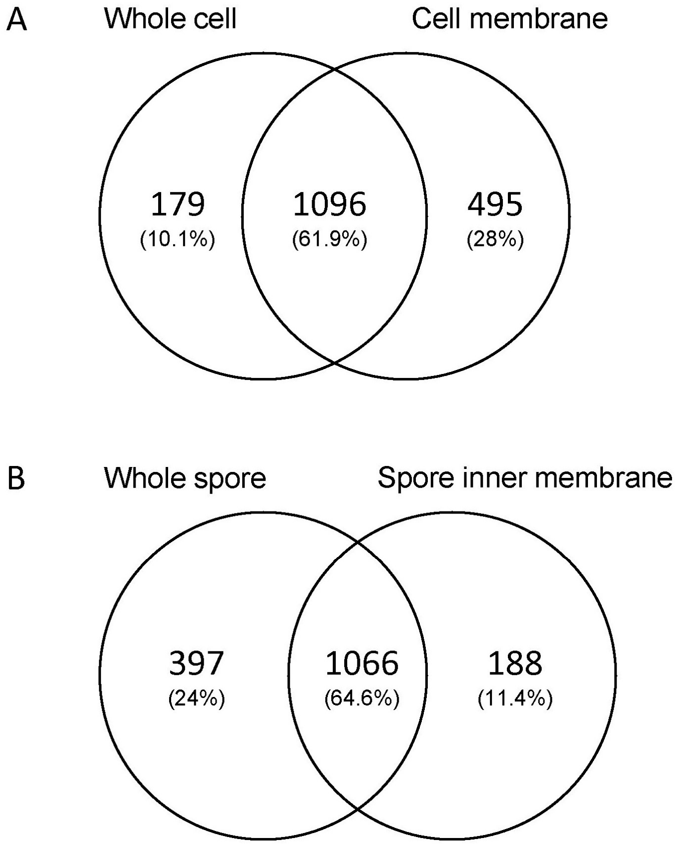

Figure S1. Venn diagrams of proteins identified in the whole cell or spore and membrane fractions. The numbers and corresponding percentages are summarized. (A) Proteins identified in Whole cell using "One-pot" method and enriched cell membrane fractions. (B) Proteins identified in Whole spore using "One-pot" method and enriched spore inner membrane fractions.
